# Columnar connectome: toward a mathematics of brain function

**DOI:** 10.1162/netn_a_00088

**Published:** 2019-07-01

**Authors:** Anna Wang Roe

**Affiliations:** Institute of Interdisciplinary Neuroscience and Technology, Zhejiang University, Hangzhou, China

**Keywords:** Primate, Cerebral cortex, Functional networks, Functional tract tracing, Matrix mapping, Brain theory, Artificial intelligence

## Abstract

Understanding brain networks is important for many fields, including neuroscience, psychology, medicine, and artificial intelligence. To address this fundamental need, there are multiple ongoing connectome projects in the United States, Europe, and Asia producing brain connection maps with resolutions at macro- and microscales. However, still lacking is a mesoscale connectome. This viewpoint (1) explains the need for a mesoscale connectome in the primate brain (the columnar connectome), (2) presents a new method for acquiring such data rapidly on a large scale, and (3) proposes how one might use such data to achieve a mathematics of brain function.

## THE COLUMNAR CONNECTOME

### The Cerebral Cortex Is Composed of Modular Processing Units Termed “Columns”

**Figure 1.**
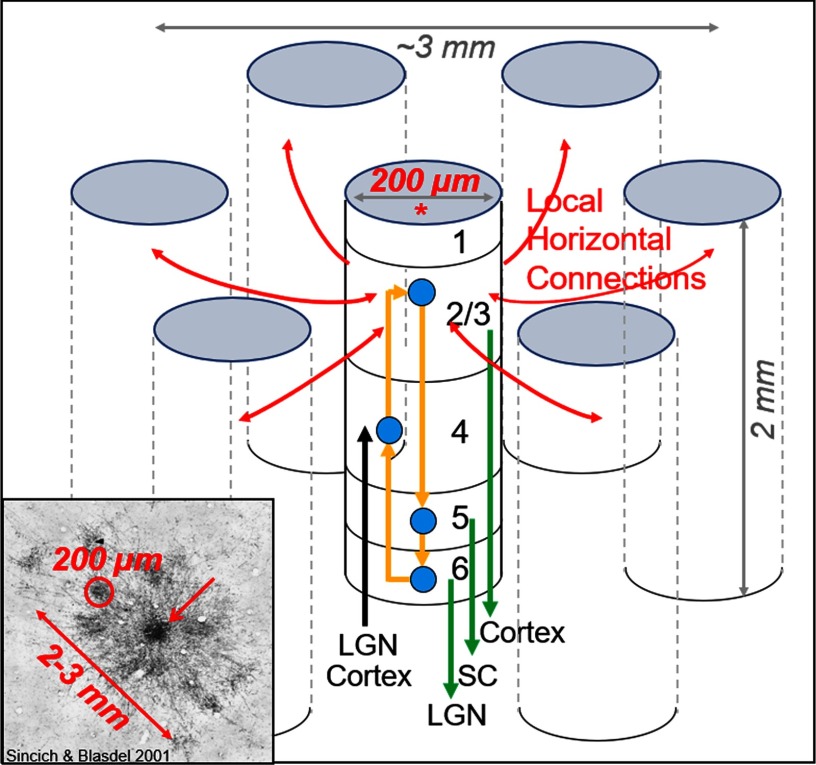
The canonical cortical column and its connections (based on nonhuman primate cortex). The cortical column (*) is a very organized input/output device, ∼200 *μ*m in size and comprising 6 layers. Layer 4 receives inputs (black arrow, e.g., from thalamus or other visual areas). Layers 2 and 3 provide outputs to other cortical columns within the same cortical area (red arrows) as well as to other cortical areas (green arrow from layer 2/3). Layers 5 and 6 provide outputs to subcortical targets (green arrows, e.g., LGN: lateral geniculate nucleus; SC: superior colliculus). All 6 layers share information via interlaminar connections (yellow arrows). Through these connections, the thousands of cells in each column share similar function; therefore, each column is functionally specific. Bottom left inset provides example of one such network (adapted from Sincich & Blasdel, 2001). Star-like arrangement of connections between columns within a local network (top down view from surface of cortex). Labeled orientation columns (∼200 *μ*m in size, one circled in red) have orientation preference similar to the injected column (red arrow). This local anatomical network embodies the concept of orientation selectivity.

In humans and nonhuman primates, the cerebral cortex occupies a large proportion of brain volume. This remarkable structure is highly organized. Anatomically, it is a two-dimensional (2D) sheet, roughly 2mm in thickness, and divided into different cortical areas, each specializing in some aspect of sensory, motor, cognitive, and limbic function. There is a large literature, especially from studies of the nonhuman primate visual cortex, to support the view that the cerebral cortex is composed of submillimeter modular functional units, termed “columns” (Mountcastle, 1997). Columns span the 2-mm thickness of cortex and are characterized by six input/output layers (laminae) linked together via interlaminar circuits (Figure 1). The tens of thousands of neurons within a single column are not functionally identical but share a common functional preference such that single stimuli maximally activate the population and produce a coherent columnar response. These coherent responses can be visualized using multiple methods, including electrophysiology (e.g., Hubel & Wiesel, 1977; Mountcastle, 1997; Katzner et al., 2009), 2-deoxyglucose (e.g., Tootell et al., 1988), optical imaging (e.g., Blasdel & Salama, 1986; Grinvald et al., 1986), and high spatial resolution fMRI methods (e.g., Cheng, 2012; Nasr et al., 2016; Li et al., 2019). More in-depth and scholarly articles about the definition and existence of the column are available (e.g., Horton & Adams, 2005; Rakic, 2008; Ts’o et al., 2009; da Costa & Martin, 2010; Rockland, 2010).

In nonvisual cortical areas, data on columnar organization is more limited (DeFelipe et al., 1986; Lund et al., 1993; Kritzer & Goldman-Rakic, 1995; Friedman et al., 2004; Gharbawie et al., 2014). However, there is accumulating evidence, as well as compelling genetic developmental (Rakic, 1988; Torii et al., 2009; Li et al., 2012), and computational reasons (Swindale, 2004; Schwalger et al., 2017; Berkowitz & Sharpee, 2018) to believe that columnar organization may be a fundamental feature throughout cortex. Pasko Rakic (2008) writes: “The neurons within a given column are stereotypically interconnected in the vertical dimension, share extrinsic connectivity, and hence act as basic functional units subserving a set of common static and dynamic cortical operations that include not only sensory and motor areas but also association areas subserving the highest cognitive functions.” For the purposes of this viewpoint, the term “column” refers to a unit of information integration and functional specificity.

### Why a Columnar Connectome Is Needed

**Figure 2.**
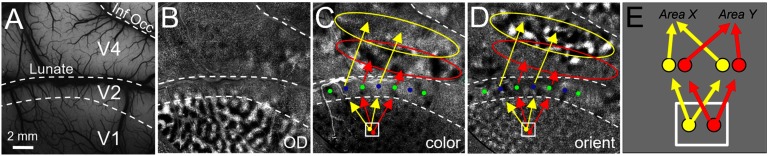
Organization of columnar connections in visual cortex. (A) Cortical surface vasculature of visual areas V1, V2, and V4 in macaque monkey brain. (B) optical image obtained by a CCD camera through window on the brain reveals ocular dominance map in V1 of macaque monkey (dark and light columns activated by left eye and right eye, respectively). (C) When monkey views a color (red/green isoluminant) grating on a monitor, this optical image reveals activated color columns (V1: dark “blobs”; V2: dark “thin stripes” indicated by green dots; V4: domains in “color band” indicated by red oval). (D) When monkey views an achromatic grating, orientation columns are revealed (vertical minus horizontal gratings). V1: orientation columns; V2: orientation domains in “thick/pale” stripes indicated by blue dots; V4: domains in “orientation band” indicated by yellow oval. Arrows are schematics of connectivity between columns in V1, V2, and V4. Red arrows: connectivity between blobs in V1, thin stripes in V2, and color bands in V4. Yellow arrows: connectivity between orientation domains in V1, thick/pale stripes in V2, and orientation bands in V4 (for review see Roe et al., 2012). (E) Yellow column projects to Area X, and Red column projects to Area Y. If connections are traced via a large anatomical tracer injection or large voxel (represented by white box), the result will show, incorrectly, that each of the yellow and red columns project to both Area X and Area Y. Note: for simplicity, feedback connections, e.g., from V4 to V2 and from V2 to V1, are not depicted.

Columns come in different flavors and have very specific connections with other columns. For example (Figure 2), in primary visual cortex (V1, dotted lines divide V1, V2, and V4), different functional columns focus on visual features such as eye specificity (ocular dominance columns, Figure 2B), color (blobs; Figure 1C: dark dots in V1 are color “blobs,” red dot overlies a blob), and orientation (orientation columns; Figure 1D dark and light domains in V1, yellow dot overlies a horizontal orientation domain). In the second visual area (V2), columns within the thin stripe (green dots) and thick/pale stripe (blue dots) types integrate columnar information from V1 to generate higher order parameters of color (thin stripes: hue), form (thick/pale stripes: cue-independent orientation response), and depth (thick stripes: near to far binocular disparity) (for review see Roe et al., 2009). Columns in V4 are hypothesized to perform further abstractions such as color constancy (Kusunoki et al., 2006), invariance of shape position and size (Rust & Dicarlo, 2010; Sharpee et al., 2013), and relative (vs. absolute) depth (Shiozaki et al., 2012; Fang et al., 2019) (for review see Roe et al., 2012).

A key aspect of cortical columns is their highly specific connections with other columns (Figure 2C and 2D, red and yellow arrows). This has been demonstrated from studies using focal injections of tracers targeted to single columns. Such studies have revealed sets of patchy connections, both intra-areal (Figure 1, inset) and inter-areal (Figure 2, arrows) (e.g., Livingstone & Hubel, 1984; Sincich & Horton, 2005; Shmuel et al., 2005; Federer et al., 2013). Column-specific connection patterns thus embody a functionally specific (e.g., orientation or color) network. However, to date, because of the demanding nature of these experiments, there are only a small number of such studies. Thus far, there has not been a method that permits systematic large-scale study of columnar connectivity. In fact, over 40 years after Hubel and Wiesel’s (1977) description of the organization of functional columns in V1, little is known about the *organization of cortical connectivity at the columnar level*. I propose that we extend the concept of the hypercolumn (all the machinery required to represent a single point in space) to the *connectional hypercolumn* (all the connections of that unit of representation).

### A New Mapping Method

The primary limitation of current methods are the following. (a) *Lack of spatial resolution*: most anatomical mapping methods employ tracer injections 2−5 mm in size. Human connectomes are based on resting-state or diffusion methods, which typically are mapped at 2−3 mm voxel resolution. These volumes (white rectangle in Figure 2E) encompass multiple columns and therefore reveal connections of a population of multiple functionally distinct columns. Since individual nearby columns can exhibit quite distinct connectivity patterns (e.g., Figure 2C and 2D: color blobs to thin stripes vs. orientation columns to pale/thick stripes), connections arising from such averages are inaccurate and misleading (Figure 2E). (b) *Slow and expensive*: traditional anatomical tract tracing typically requires 2 to 3 weeks for tracer transport, animal sacrifice to acquire tissue, and time-consuming weeks to map label locations and 3D reconstruction. (c) *Not large scale*: anatomical studies are limited to several tracers, and therefore the connections of only a handful of nodes can be studied in any single brain. Other methods such as electrophysiological stimulation with fMRI mapping have elegantly revealed networks underlying specific behaviors (e.g., Tolias et al., 2005); however, electrical methods can suffer from current spread, leading to lack of spatial specificity, as well as inability to map local connections because of signal dropout near the electrode. Optogenetic stimulation with fMRI mapping is a powerful cell-type specific approach (e.g., Gerits et al., 2012); however, in primates, it takes weeks for viral expression and has thus far been limited by the small number of transfected nodes, making large-scale mapping of connections in the primate brain challenging. (d) *Correlation based functional connectivity*: fMRI BOLD signal correlation (resting state studies) noninvasively probes networks in human and animal brains, but are limited to inference about correlation rather than connectivity. Such limitations also exist with neurophysiological cross-correlation studies of spike timing coincidence.

To overcome some of these limitations, we have developed a new rapid in vivo mapping technique. This method combines an optical stimulation method, termed *pulsed infrared neural stimulation* (INS), with high-field fMRI (Xu et al., 2019a). INS is a method that uses pulsed trains of light (1,875 nm) to induce heat transients in tissue (Wells et al., 2005; Cayce et al., 2014; Chernov & Roe, 2014). Although the mechanism underlying INS is still under study, the leading theory is that the heat transients lead to membrane capacitance change, which leads to induction of neuronal firing. When INS is delivered with a fine fiber optic (e.g., 200-*μ*m diameter) to the cerebral cortical surface, the light distribution is highly focal (roughly the same diameter as the fiber optic) and roughly ∼300 *μ*m in penetration depth; this penetration reaches cells in the superficial layers (2/3) and apical dendrites of pyramidal cells in deep layers (5/6). Such neuronal activation, in turn, is propagated to downstream neurons at connected sites. Similar activation can be achieved by inserting the fiber optic into deep brain sites (Xu et al., 2019b). The resulting BOLD responses associated with this activation constitutes a map of cortico-cortical connectivity arising from a single site. For example, when INS is applied to area 17 with fine 200-*μ*m optic fibers in a high-field MRI, INS evokes focal activations in areas 18 and 19, the ipsilateral LGN, and contralateral 17/18 (Xu et al., 2019a). This activation is robust, intensity dependent, and safe for long-term use (Chernov et al., 2014). One can also use high-resolution mapping to examine BOLD activation in different cortical lamina. This reveals local connections that distinguish between feedforward (layer 4 activation) and feedback (superficial and deep laminae) connections (Xu et al., 2019a). Importantly, as no animal sacrifice is required for large dataset collection, the brain can be probed systematically with fiber optic bundles applied to windows on the brain, across multiple sessions. It is also compatible with other imaging, electrophysiological, and behavioral methods for multimodal dataset correlation (Chernov & Roe, 2014; Xu et al., 2019a).

## COLUMNAR MOTIFS

**Figure 3.**
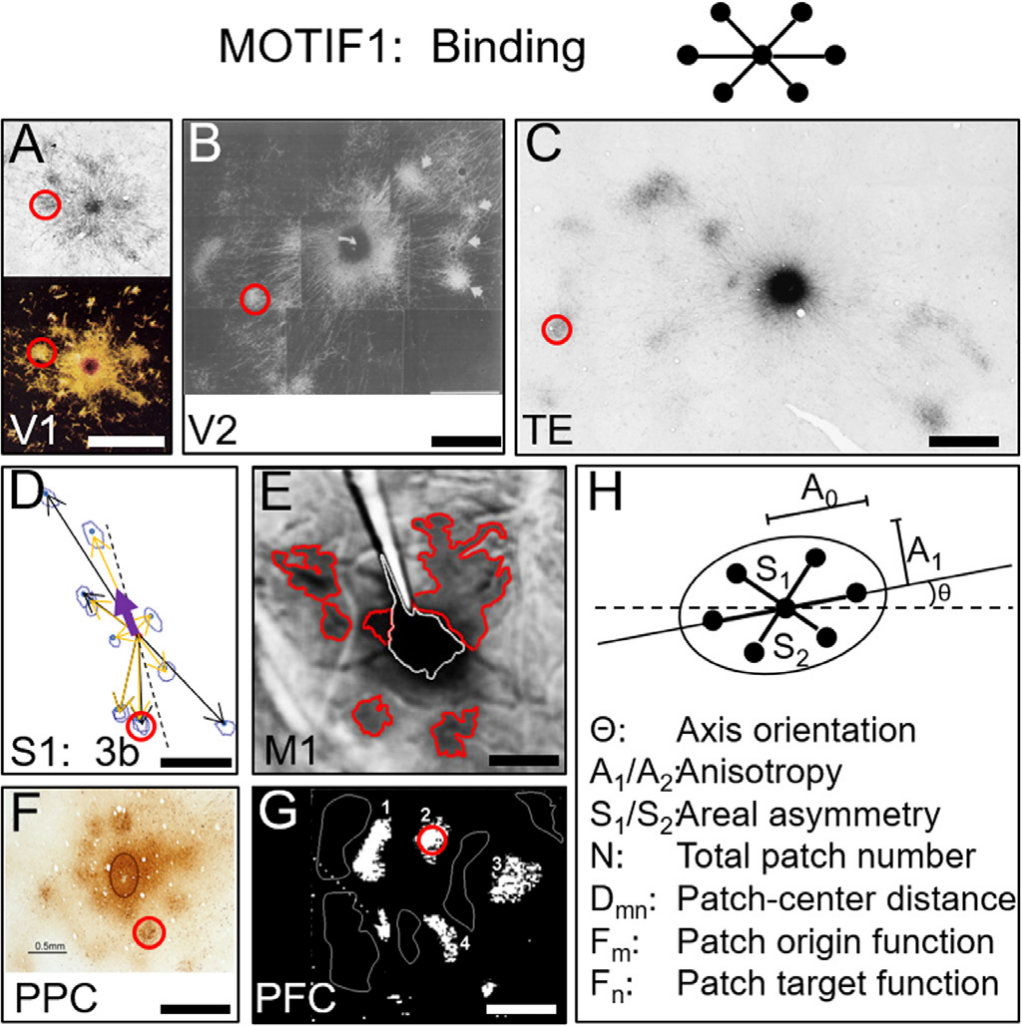
Examples of Intra-areal connection motifs. Motif1: Intra-areal circuits serve binding function and display “star-like” pattern (cartoon at top). This is exemplified by anatomical tract tracing. Labeled patches are 200 *μ*m in size (red circles). (A) V1, top: Sincich & Blasdel, 2001; bottom: Livingstone & Hubel, 1984. (B) V2, Malach et al., 1993. (C) TE, inferotemporal; Tanigawa et al., 2005. (D) S1; Pálfi et al., 2018, Wang et al., 2013. (E) M1, motor; Gharbawie et al., 2014. (F) PPC, parietal; Stepniewska et al., 2016. (G) PFC, prefrontal; Sawaguchi, 1994. (H) Motif parameters.

The long-standing notion that cortical columns perform common functional transformations would be further bolstered by the presence of columnar connectivity *motifs*. The existence of motifs would suggest that there are indeed common modes of information distribution and integration. The true value of such motifs is the possibility of identifying a small set of general motifs to characterize cortical function (Figure 3). This would provide basis vectors for constructing biological representation of information.

One could wonder why it matters what the underlying anatomical motif is. For some computational neuroscientists, it matters not what the brain circuit is, as long as a circuit can achieve the desired functional output. However, it is a fact that our brain contains organized anatomical constructs, and these constructs perform some pretty sophisticated functions that so far have been difficult to mimic with other architectures. Although the brain may not be the only architecture capable of intelligent behavior, perhaps it is one optimal architecture given existing biological and physical constraints.

Below, I present two possible motifs: an intra-areal motif (Motif1, binding) and an interareal motif (Motif2, transformation). The basis for these motifs comes from prevous studies of mesoscale anatomical connections.

### Motif1: Intra-Areal Networks (Binding)

As shown in Figure 3, many cortical areas contain striking local “star-like” topologies in which a central column is linked to nearby columns of similar functional preference. The columns within these networks are typically 200–300 *μ*ms in size (red circles) (Amir et al., 1993). A few examples from the literature illustrate the ubiquity of these constructs; areas include visual (A–C), sensorimotor (D–E), parietal (F), and prefrontal (G) areas. In V1, injection of tracer into an orientation column labels nearby columns of similar orientation preference, thereby forming an orientation selective network (Figure 3A, top). Color blobs in V1 also form star-like networks (Figure 3A, bottom). In V2, color, form, and disparity columns lie within separate functional streams but are linked via interstripe connections to form single multifeature networks (Figure 3B). Similar networks are seen in temporal cortex (Figure 3C). In area 3b of somatosensory cortex, columns subserving digits D1 to D5 are linked in interdigit networks and are hypothesized to underlie sensory co-activation during power grasp (Figure 3D). Other star-like networks are observed in primary motor (Figure 3E), posterior parietal (Figure 3F), and prefrontal (Figure 3G) areas.

In Motif1, the central node integrates inputs from a subset of surrounding nodes, thereby performing a “binding” function. In some areas, nodes of similar functionality (e.g., V1; Ts’o et al., 1986) are linked, whereas, in others, nodes bind different modalities into a coherent multimodal representation (e.g., orientation, color, and disparity via interstripe connections in V2; Levitt et al., 1994). Note that opposing networks of complementary function are similarly “bound” via inhibitory relationships. Together, these interdigitated networks underlie push-pull functions (e.g., white vs. outlined domains in Figure 3G; Cayce et al., 2014; Chernov et al., 2018; cf. Sawaguchi, 1994; Weliky et al., 1995; Sato et al., 1996; Toth et al., 1996).

The specific characteristics of these networks (such as overall size, number of nodes, axis, anisotropy; Figure 3H) may be tailored to the functional/computational purpose of each area and may be influenced by more global factors, such as size of the cortical area or by the number of other linked cortical areas. These possibilities remain to be explored and tested via mesoscale connectome data.

### Motif2: Inter-Areal Networks (Transformation)

This motif captures the anatomical connections underlying transformations from one cortical area to another. It is well known that representation becomes increasingly abstract with cortical hierarchy. However, it is unknown whether the connections from one area to the next that mediate functional transformations are in any way systematic or standard. If so, one might be able to systematize the transformations into functional classes, potentially reducing all cortico-cortical projections into a small set of functions. In addition to providing a mathematical way to represent the brain concisely, it would provide insight into the function of areas for which there is as yet little understanding.

The idea that there are common functional transformations derives from studies in early sensory cortical areas. *Vision*: From studies that simultaneously monitor responses of V2 and V1 function to single “illusory” stimuli, we have shown that neurons in V2 domains respond to the illusory aspect, whereas neurons in V1 respond to the “real” aspect (Roe, 2009, for review). This “real-to-illusory” higher order transformation must be mediated by the anatomical connectivity between V1 and V2. Specifically, we observe establishment of modality-specific higher order properties in different stripes of V2: (a) *thin stripes*: color representation in V1 blobs, which is dominated by red-green/blue-yellow axes, transforms to a multicolor map of hue columns in V2 (Conway, 2001; Xiao et al., 2003); (b) *thin stripes*: luminance encoding in V1 transforms to brightness encoding in V2 (Roe et al., 2005); (c) *thick/pale stripes*: encoding of simple contour orientation transforms to higher order cue-invariant orientation representation in V2 (Rasch et al., 2013); (d) *thick stripes*: simple motion direction detection transforms to the detection of coherent motion in V2 (useful for figure-ground segregation, Peterhans and von der Heydt) and to motion contrast defined borders (Hu et al., 2018); and (e) *thick stripes*: segregated representation of left and right eyes in V1 to maps of near-to-far binocular disparity columns in V2 (Chen et al., 2008, 2017). *Touch*: In a similar vein, in somatosensory cortex, integration of tactile pressure domains in area 3b (Friedman et al., 2004) are hypothesized to generate motion selectivity domains in area 1 (Pei et al., 2010; Wang et al., 2013; Roe et al., 2017). These modality-specific transformations could be achieved by integrating across multiple unimodal inputs in V1 and in S1.

**Figure 4.**
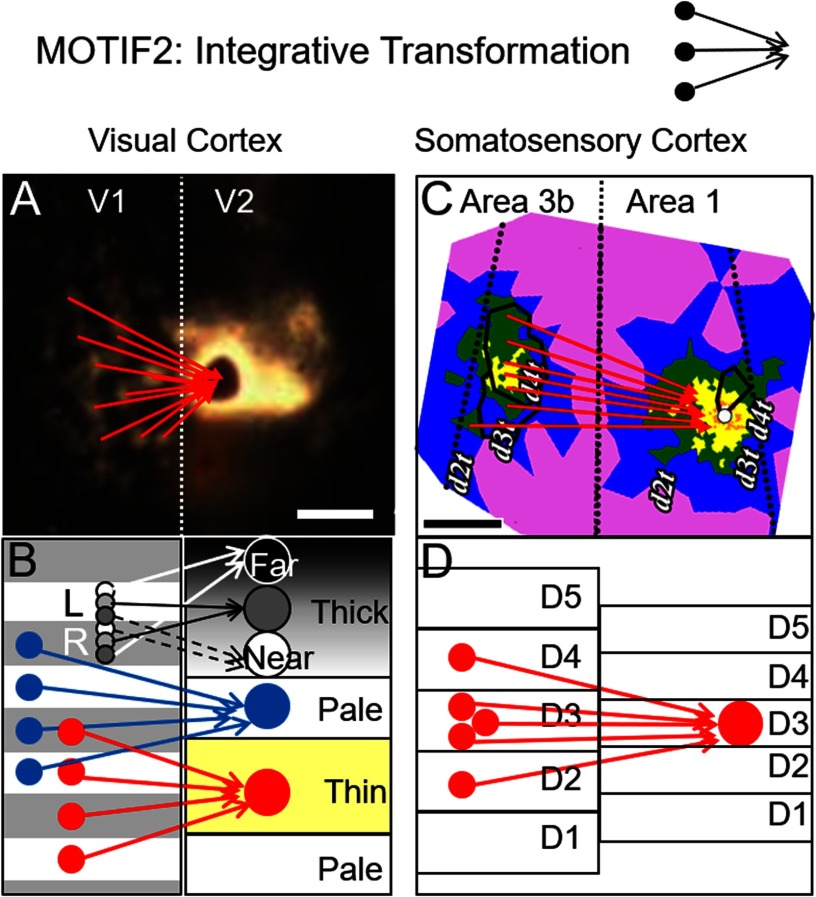
Examples of Interareal connection motifs. Motif2: interareal circuits serve to transform representation by integration of inputs from multiple columns. (A) Image of labeled blobs in V1 following injection of tracer in V2 (Sincich & Horton, 2005). Red arrows: interblob inputs from V1 converge onto a single pale stripe in V2. Vertical dotted line: V1/V2 border. (B) This motif is observed from (red) V1 blobs to V2 thin stripes, (blue) V1 interblobs to V2 pale/thick stripes, and (grays) from V1 ocular dominance columns to V2 thick stripes. (C, D) Red arrows: converging digit tip inputs from Area 3b to Area 1 in somatosensory cortex (Wang et al., 2013).

Remarkably, common anatomical motifs underlie these functional computations. Injection of tracer into V2 labels multiple columns in V1 (Figure 4). These motifs are observed following injections into thin stripes (Figure 4B, red), pale stripes (Figure 4B, blue), and thick stripes (Figure 4B, grays). Similarly, injection of tracer into a single digit location in area 1 labels multiple (presumed) columns in area 3a (Figure 4C and 4D). Although it remains to be seen whether such proposed transformations are also found in other sensory, motor, and cognitive systems, identification of such common motifs (along with characteristic integration size, number, and functional type) would be important for generating an understanding of how the anatomy of brain connections leads to and limits brain function.

## SOME THOUGHTS ABOUT MATHEMATICAL FORMULATION

There is a very large gap between what is known about cortico-cortical connection patterns and what we need to know to guide concepts about mappings in mathematical terms. Ultimately we should like to know what are the organizational principles underlying cortical connection patterns. The work of investigators such as Obermayer and Blasdel (1993), Goodhill and Cimponeriu (2000), Carreira-Perpiñán et al. (2005), Swindale (2004, 2007), and others have beautifully demonstrated that the arrangement of multiple maps within an area arises largely through maximizing parameters of continuity and coverage. Similar constraints need to be identified and modelled for cortico-cortical connectivity. Progress on this front will be greatly aided by availability of data on columnar connectivity. There are numerous efforts to characterize connectomes by using mathematical topology (cf. Sporns, 2011). However, greater attention needs to be focused at the columnar scale.

Here, I propose one possible view of modelling cortical connections. Although representation of some parameters is continuous (e.g., continuously shifting orientation preference), a “binned” representation is valid from the view of functional organization; that is, continuity gets broken up into columnar representations by the constraint of mapping multiple parameters within a single sheet (Obermayer & Blasdel, 1993; Swindale, 2004).

### Matrix Mapping

**Figure 5.**
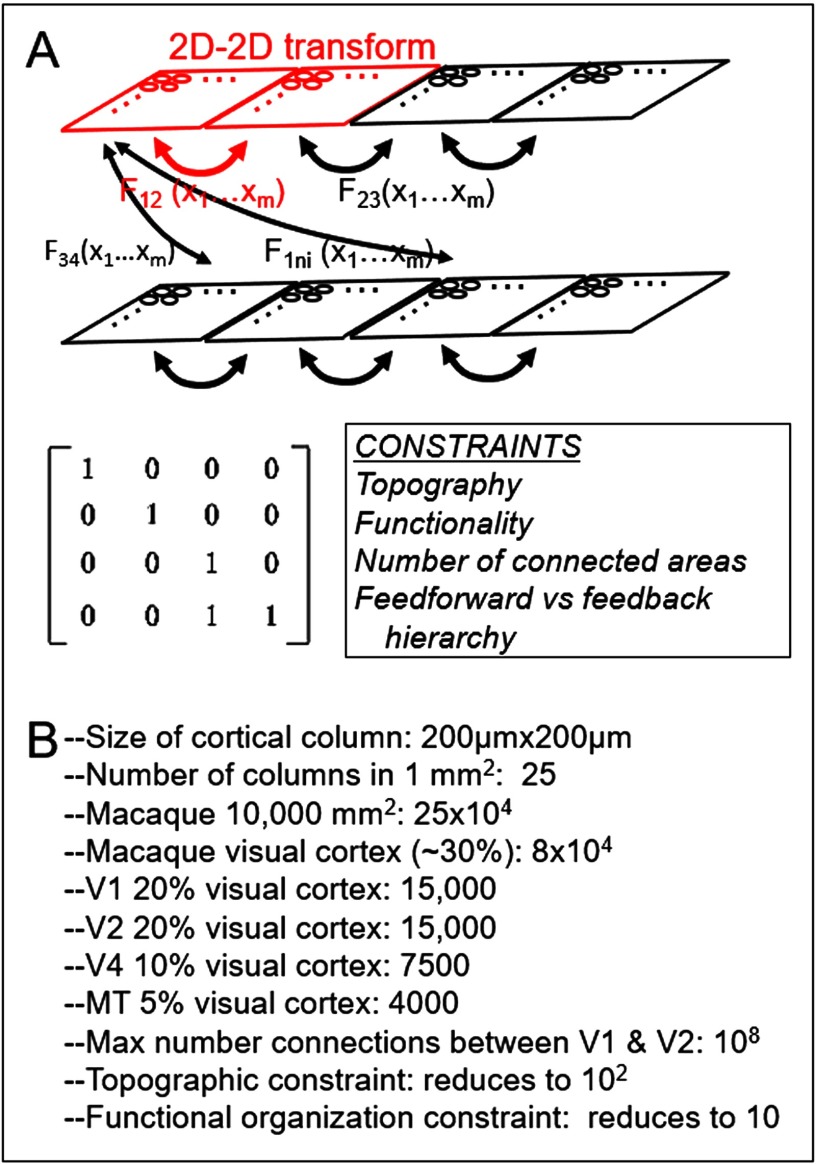
2D-to-2D matrix representation of cortico-cortical mapping. (A) Each cortical area (rectangle) is represented as a 2D array of columns (circles). Red outlined portion: mapping between a single pair of areas. This 2D-2D mapping can be expressed as a matrix transformation (below) with identified constraints such as topography and functional selectivity. The hope is that one can reduce the mappings to a small number of motifs (Fij). (B) Some rough numbers used to suggest that computation is manageable.

If one views the cortical sheet as a 2D array of columns, then connections between cortical areas can be viewed as 2D-to-2D mappings (Figure 5, red outlined portion). The challenge to characterize connectional motifs may then be expressed as identifying generalized 2D-2D matrix mappings that govern how two cortical areas connect (Figure 5A, F_*ij*_). For any pair of areas, such mappings would be constrained by anatomical and functional constraints such as continuity (topography) and functional specificity. One additional constraint to be considered might include the number of areas with which a single area directly connects (typically this is a small number). Once an anatomical scaffold is constructed, column-specific feedforward and feedback modulation could be implemented to model circuit dynamics. From such treatment of connectivity, general patterns of mappings (motifs) may emerge. Although the problem may, at first glance appear formidable, in my view, constraints of anatomical architecture dictate that each cortical area maps only a few (e.g., 3 to 4) key parameters in a continuous and complete fashion. I make an argument below that this matrix representation may help reduce the complexity.

### Computational Feasibility

A few back-of-the-envelope estimates suggest that this mapping problem may be computationally manageable (Figure 5B). These estimates are based on published neuroanatomical studies and inferred order-of-magnitude calculations. We use 200 *μ*m as the dimension of a cortical column in macaque monkey (size of orientation domains and blobs in V1); each mm^2^ then contains 25 columns. The macaque neocortex has an area of about 10,000–15,000 mm^2^, roughly a third of which is visual cortex (cf. Van Essen et al., 1984; Purves & LaMantia, 1993; Sincich et al., 2003). This means that there are on the order of 25 × 10^4^ columns total and about (a third) 8 × 10^4^ columns in visual cortex. [Note: in humans, although visual cortex is 3 times as large, due to larger column size (roughly double), there are a comparable number of columns (Horton & Hedley-Whyte, 1984; Adams et al., 2007).] Out of this total of 80,000 columns, V1, V2, and V4 occupy roughly 20%, 20%, and 10% of visual cortical area, or 15,000, 15,000, and 7,500 columns, respectively (Weller et al., 1983; Sincich et al., 2003). This is likely an underestimate as it assumes square columnar packing.

If all columns in V1 mapped to all columns in V2, the number of single node-to-single node connections would be on the order of 10^8^ (15,000 × 15,000) (Figure 5B). From neuroanatomical studies, we estimate that a single node in V1 connects to on the order of 10 nodes in V2 (Livingstone & Hubel, 1984; Federer et al., 2013; Sincich et al., 2010). This small number is due to (a) constraints of topography: a single point in visual space takes up roughly 2 mm of cortical space (Hubel & Wiesel, 1977), or 4 × 25 = 100 columns (10^2^); and (b) constraints of functional specificity (Figure 4), which further reduces this number by a fifth or a tenth the number of nodes from a single topographic point. As an example, if a full 180° cycle of orientations is represented in 1-mm distance (Hubel & Wiesel, 1977) or 5 (200 *μ*m-sized) columns, then within a 1-mm^2^ area there may be 5 out of 25 columns that represent a single orientation. This reduces the total number of target nodes from 100 to 10–20 or on the order of 10. This potentially reduces a problem that is on the order of 10^8^ to 10. Such a 1:10 convergence/divergence could underlie a basic architectural motif of interareal connectivity, or an “interareal connectional hypercolumn.”

Such connectional hypercolumns may be replicated at a higher level, albeit with distinct convergence/divergence ratios and functional constraints. For example, from V2 to V4, the number of connections decreases (15,000 × 7,500, or 10^7^), producing a correspondingly smaller cortical area (V4 is roughly half the size of V2). An area such as MT, which is only 5% the size of V1, is likely to have a greater convergence/divergence ratio from V1 to MT; this would be consistent with the large receptive field sizes and comparatively spatially broad integrations for computation of motion direction. Thus, interareal connectivity would be specified by parameters such as unique topographical constraints, convergence/divergence ratios, and functional selectivities. This set of relationships would be represented as a set of connectional hypercolumns: F_1_ (x_1_…x_*m*_) … F_*n*_(x_1_…x_*m*_). Solving how multiple connectional hypercolumns mutually constrain the entire set of mappings will lead to a representation of total cortical connectivity in a brain.

### Columnar Organization Constrains the Global Network

Thus far, topological treatment of brain networks has been based on parameters such as connection number, connectional distance, routing efficiency (e.g., Young, 1993; Avena-Koenigsberger et al., 2019; Chaudhuri et al., 2015). These approaches have provided important advances in our understanding of brain networks and information flow at an areal level (typically with each cortical area treated as a single node). However, what is lacking is the concept of spatial location within each area. That is, the X, Y coordinate in the matrix defines a node’s topographical location and its functionality. Each node’s position is not independent of another nodes position; neighbors constrain neighbors, and this impacts the organization of the global network. Studies that address issues of multiscale constraints (e.g., Jeub et al., 2018) could incorporate such spatial information to further specify the resulting architecture.

## CONCLUSION

To summarize, I have described a column-based view of the cerebral cortex and presented a new methodology (INS-fMRI) to map column-based networks *in vivo*. I suggest that acquisition of such large-scale connectivity data may demand new ways of representing cortical networks (e.g., 2D-2D matrix transformations). From such data, patterns or motifs of cortical connectivity may emerge, and give rise to basic connectivity units termed *connectional hypercolumns*.

My goal in this viewpoint is to encourage the connectomics field to capture columnar connectivity. New representations and mathematics need to be developed for multidimensional treatment of nodes, one that incorporates the spatial and functional relationships between neighboring nodes. Such representations may simplify the apparently complex connectional relationships in the global network. Although we do not yet have enough columnar data to do this on a large scale, one could start by using available data to generate prototype solutions. I list a few questions here to motivate future studies. For a given cortical area, how does the number of directly connected areas affect motif architecture? How do the number of total areas affect the total possible mappings? Is there a general solution to the mappings of smaller brains with fewer cortical areas versus larger brains with many? What aspects of cortical architecture produce and simultaneously constrain our behavioral repertoire? Can one design mappings to generate alternative artificial intelligences?

Ultimately, I envision a general connectional theory of brain function, complete with a system of theorems, derivations, and corollaries (Sporns et al., 2000; Sporns, 2011). Such a rule-based representation will lead to new understandings of brain construction and brain evolution, and will inform our understanding of biological intelligence as well as bio-inspired artificial intelligence.

## ACKNOWLEDGMENTS

Thanks to Charles Gilbert and Akshay Edathodathil for useful discussions.

## FUNDING INFORMATION

Anna Wang Roe, National Natural Science Foundation of China (http://dx.doi.org/10.13039/501100001809), Award ID: 81430010. Anna Wang Roe, National Natural Science Foundation of China (http://dx.doi.org/10.13039/501100001809), Award ID: 31627802. Anna Wang Roe, National Hi-Tech Research and Development Program Grant, Award ID: 2015AA020515.

## TECHNICAL TERMS

Column: A modular functional unit of cerebral cortex.
Optogenetics: A method of controlling neurons by light by viral transfection of rhodopsin proteins.
Mesoscale: The submilleter scale, columnar scale.
Connectome: A complete map of neural connections in the brain, or its “wiring diagram.”
